# CLUSTOM-CLOUD: In-Memory Data Grid-Based Software for Clustering 16S rRNA Sequence Data in the Cloud Environment

**DOI:** 10.1371/journal.pone.0151064

**Published:** 2016-03-08

**Authors:** Jeongsu Oh, Chi-Hwan Choi, Min-Kyu Park, Byung Kwon Kim, Kyuin Hwang, Sang-Heon Lee, Soon Gyu Hong, Arshan Nasir, Wan-Sup Cho, Kyung Mo Kim

**Affiliations:** 1 Microbial Resource Center, Korea Research Institute of Bioscience and Biotechnology, Daejeon, Republic of Korea; 2 Department of Bio-Information Technology, Chungbuk National University, CheongJu, Republic of Korea; 3 Department of Business Data Convergence, Chungbuk National University, CheongJu, Republic of Korea; 4 BioNano Health Guard Research Center, Korea Research Institute of Bioscience and Biotechnology, Daejeon, Republic of Korea; 5 Division of Polar Life Sciences, Korea Polar Research Institute, Incheon, Republic of Korea; 6 Department of Biosciences, COMSATS Institute of Information Technology, Islamabad, Pakistan; 7 Department of Management Information Systems/BK Plus Team, Chungbuk National University, CheongJu, Republic of Korea; 8 Department of Bioinformatics, University of Science and Technology, Daejeon, Republic of Korea; CNRS UMR7622 & University Paris 6 Pierre-et-Marie-Curie, FRANCE

## Abstract

High-throughput sequencing can produce hundreds of thousands of 16S rRNA sequence reads corresponding to different organisms present in the environmental samples. Typically, analysis of microbial diversity in bioinformatics starts from pre-processing followed by clustering 16S rRNA reads into relatively fewer operational taxonomic units (OTUs). The OTUs are reliable indicators of microbial diversity and greatly accelerate the downstream analysis time. However, existing hierarchical clustering algorithms that are generally more accurate than greedy heuristic algorithms struggle with large sequence datasets. To keep pace with the rapid rise in sequencing data, we present CLUSTOM-CLOUD, which is the first distributed sequence clustering program based on In-Memory Data Grid (IMDG) technology–a distributed data structure to store all data in the main memory of multiple computing nodes. The IMDG technology helps CLUSTOM-CLOUD to enhance both its capability of handling larger datasets and its computational scalability better than its ancestor, CLUSTOM, while maintaining high accuracy. Clustering speed of CLUSTOM-CLOUD was evaluated on published 16S rRNA human microbiome sequence datasets using the small laboratory cluster (10 nodes) and under the Amazon EC2 cloud-computing environments. Under the laboratory environment, it required only ~3 hours to process dataset of size 200 K reads regardless of the complexity of the human microbiome data. In turn, one million reads were processed in approximately 20, 14, and 11 hours when utilizing 20, 30, and 40 nodes on the Amazon EC2 cloud-computing environment. The running time evaluation indicates that CLUSTOM-CLOUD can handle much larger sequence datasets than CLUSTOM and is also a scalable distributed processing system. The comparative accuracy test using 16S rRNA pyrosequences of a mock community shows that CLUSTOM-CLOUD achieves higher accuracy than DOTUR, mothur, ESPRIT-Tree, UCLUST and Swarm. CLUSTOM-CLOUD is written in JAVA and is freely available at http://clustomcloud.kopri.re.kr.

## Introduction

Microorganisms constitute a vital component of the biosphere. They are remarkably abundant and occupy many kinds of environments including various human body sites [1]. Microbes play crucial roles in ecosystems. They are involved in nitrogen fixation, utilized in the medicine and food industry, cause numerous diseases, and are a rich source of new genes for other organisms. Despite having been extensively studied for decades, many aspects of microbial ecology and evolution still remain unexplored. Since the vast majority of microbes cannot be cultured, it is desirable to analyze microbial diversity directly from environmental samples. Recent advances in next-generation sequencing (NGS) have enabled generation of large amount of 16S ribosomal RNA (rRNA) sequence data recovered directly from a variety of environmental samples. Although the data provide a unique opportunity to deeply examine microbial diversity and community structure, its efficient handling remains a bioinformatics challenge.

The first step in the analysis of microbial samples recovered from various environments is pre-processing, which includes trimming tag sequences, filtering out the low-quality reads, removing chimera errors and dereplication [2]. Pre-processing is followed by clustering 16S rRNA sequences into operational taxonomic units (OTUs). Since the number and size of OTUs are obligatory indicators of the richness (e.g., number of different species) and evenness (relative abundance of individual species across taxonomic groups) of microbial taxa present in the samples [3, 4], sequence clustering is regarded as one of the most important and influential steps in analyzing environmental data. Sequence clustering is effectively a taxonomy-independent (or *de novo*) approach that can group microbial taxa even in the absence of reference databases of known organisms [5]. Moreover, the use of representative OTUs greatly reduces the computational time required in the downstream analysis of extensive data generated by high-throughput sequencing platforms. Several algorithms have therefore been developed for accurate and fast clustering of sequences into OTUs. Some well-known options include DOTUR [6], ESPRIT [7], Cd-hit [8], UCLUST [9], mothur [10], CLUSTOM [11] and Swarm [12]. In a recent comparative study, we showed that CLUSTOM gave superior clustering performance and guaranteed both optimal accuracy and speed relative to its contemporaries [11]. Specifically, the clustering accuracy of CLUSTOM was similar to DOTUR but better than other hierarchical clustering algorithms. Importantly, CLUSTOM that uses *k*-mer thresholds in clustering sequences outperformed DOTUR and mothur in terms of computational time even though the computational complexity of the three programs is basically *O*(*N*^*2*^). While both Cd-hit and UCLUST (greedy heuristic clustering algorithms of complexity *O*(*N*^*1*.*2*^)) were faster than CLUSTOM, their accuracy did not reach desirable levels [11]. Therefore, CLUSTOM should be considered the method of choice to efficiently and accurately cluster 16S rRNA sequences into OTUs, and importantly, is based on the natural phenomenon of prokaryotic species divergence, unlike other algorithms [11]. However, the original version of CLUSTOM relied on the CPU and memory resources of a single computational node, which limited its use to small-scale datasets (generally up to 300K reads). To overcome this limitation we present CLUSTOM-CLOUD, a significant upgrade to CLUSTOM, which can run in both the distributed and cloud-computing environments and can be conveniently scaled to process over one million 16S sequences.

Current distributed computing techniques such as Message Passing Interface (MPI) and MapReduce of the Hadoop ecosystem are widely used to analyze large-scale sequence data in bioinformatics fields. MPI has been in use for some time and offers portability and reliable performance [13]. However, it is difficult to make application durable of fault tolerance which means the ability to recover from one or more component failures in a manner with a transparent or application-driven mechanism. In turn, MapReduce of the Hadoop offers high scalability and fault tolerance and is relatively easier to use [14–16]. However, it suffers from occasional performance degradation and slow speed due to I/O bottlenecks between the CPU and the hard disk [17, 18]. In addition, Hadoop MapReduce-based programs demand specific installations and settings similar to MPI, which can be difficult to implement and time-consuming. Because of these limitations, a different distributed computing approach such as the ‘In-Memory Data Grid (IMDG)’ technology needs to be considered. This distributed approach has recently become popular in industrial and information science fields to handle “big data”. Some well-known applications include online banking, risk analysis, trading systems, e-commerce, and online gaming [19]. The IMDG data structure can store terabytes of data in Random Access Memory (RAM) shared by multiple nodes. It provides three key advantages over MPI and Hadoop MapReduce: (i) processes data much faster because all data can be stored and accessed in memory, (ii) can be relatively easily scaled and executed without any complicated installation, and (iii) is easy to implement for both parallel and distributed computing systems.

To assess the speed performance of CLUSTOM-CLOUD, we prepared four sequence datasets of various sizes (50 K, 100 K, 150 K, and 200 K) from 16S rRNA data of microbial communities sequenced from different human body sites [1] exhibiting *high*, *intermediate*, and *low* microbial complexity (see Methods). To demonstrate the scalability of CLUSTOM-CLOUD, we analyzed one million randomly sampled sequences from the above-mentioned datasets by gradually increasing the computing nodes on the Amazon Elastic Compute Cloud (EC2) computing environment (20, 30, and 40 nodes). Comparative accuracy evaluation of clustering programs was also conducted using 16S rRNA sequences of a mock community. The speed and accuracy comparison results revealed that implementation of IMDG technology and its additional enhancements such as sequence dereplication and *k*-mer transformation method (read below) in CLUSTOM-CLOUD yielded speedier processing of large sequence datasets than its ancestor CLUSTOM while maintaining the high accuracy. CLUSTOM-CLOUD is platform-independent and can be conveniently scaled and upgraded to keep pace with the continuous rise of biological sequence data.

## Methods

### System architecture

**Fig 1:**
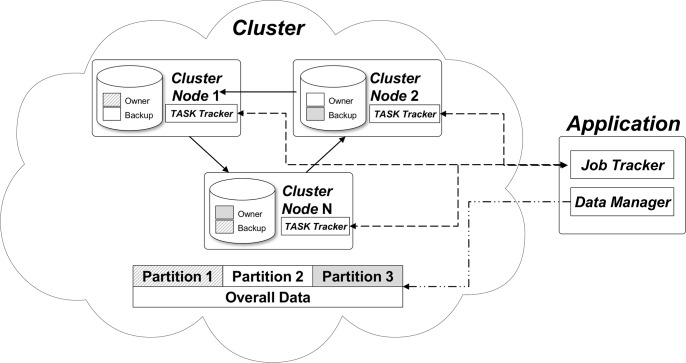
System architecture. CLUSTOM-CLOUD consists of *Application* and *Cluster* units. *Application* is composed of *Job Tracker* and *Data Manager*. *Job Tracker* assigns *Task Tracker* to each *Cluster Node* in *Cluster* and checks its status. *Task Tracker* processes a distributed task in parallel using multi-threads. Data manager manages processed results and generates clustering results. *Cluster* is a set of *N*-nodes, which are unified by IMDG. *Cluster* is composed of *Cluster Node* and *Task Tracker*. A part of RAM in each *Cluster Node* is assigned to IMDG data structure and backup area.

OTUs are inferred from the genetic distance between each pair of 16S rRNA sequences. While the precise calculation of genetic distance requires exhaustive implementation of dynamic programming such as the Needleman-Wunsch (NW) algorithm, determining optimal alignments between millions of possible sequence pairs can be computationally intensive. While the heavy computational time is difficult to reduce, we can significantly reduce the wall clock time by building a high-performance distributed computing system. Following this rationale, we adopted the *k*-mer transformation method and sequence dereplication steps (read below) to significantly reduce the computational time while also considering network overhead effects by finding optimal data granularity. Moreover, the majority of raw and intermediate data needs to be stored in memory (i.e., IMDG data structure) rather than the hard disk of individual nodes for faster execution. In consideration of these points, CLUSTOM-CLOUD has utilized a hybrid architecture comprising both peer-to-peer (useful for high-performance distributed computing) and client-server (appropriate because most data needs to be stored in memory) cluster approaches [20]. The hybrid architecture consists of two components: *Cluster* and *Application* (Fig 1).

*Cluster* is a collection of *N*-nodes that share the computational task of deriving OTUs and determining sequence clusters. To support IMDG, each *Cluster Node* partially stores overall data in the ‘owner’ area of RAM (Fig 1), referenced by the ‘key-value’ data structure (read below). To avoid data loss resulting from the unexpected shutdown of some nodes, each node has an additional ‘backup’ area linked to another node (solid lines in Fig 1). The owner and backup constitute a single data partition [20] and a similar design is implemented across *N*-nodes to store the entire dataset directly into memory. In turn, *Task Tracker* is a logical unit for processing requested tasks. It contains definitions of operations, information about the required number of threads, and the range of sequences to be processed by each node. *Task Tracker* processes a distributed task in parallel using multi-threads and is assigned to each node by the *Job Tracker* of *Application*.

The *Application* unit activates the *Cluster* upon user request. It is composed of *Job Tracker* and *Data Manager*. *Job Tracker* assigns *Task Tracker* to each *Cluster Node* (dotted lines in Fig 1) and routinely checks its status. In case of any malfunction, it can automatically reassign *Task Tracker* to another node, thus continuing the operational chain. Although the IMDG data structure provides functionality for checking node heartbeat, it does not support reassigning a failed task to another node. Therefore, *Job Tracker* is a crucial component of the architecture to support the high availability of CLUSTOM-CLOUD. In turn, *Data Manager* integrates partial results from multiple *Task Trackers* and generates initial and final clustering results (Fig 1, double dashed line). It also clears temporary data from memory to ensure maximum availability of resources during all stages of processing.

### Clustering workflow

**Fig 2:**
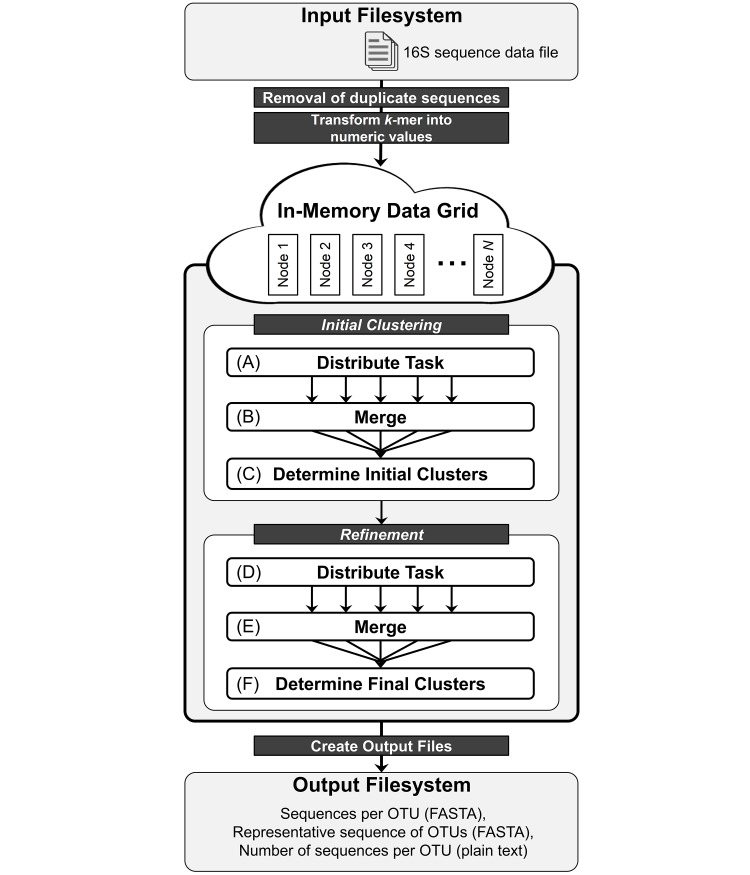
Schematic diagram of clustering workflow. 16S rRNA sequences in FASTA format are provided as input. Each input file, already checked for low-quality and chimera errors, is pre-processed by the removal of duplicates and transformation of *k*-mer into numeric values. A fixed number of sequence pairs are distributed to each cluster node for *k*-mer (initial) and NW (refinement) distance calculation. Processed results are merged upon completion of each unit task. Clusters are determined based on criteria described previously [11] and in text. Output files are created and data are cleared from memory.

The workflow of the previous version CLUSTOM consisted of three major steps: *k-mer threshold determination*, *initial clustering* and *refinement*. In the *k-mer threshold determination* step, CLUSTOM randomly samples a subset of sequences from a given 16S input dataset. Next, *k*-mer and NW distances between all possible sampled sequence pairs are calculated to determine the *k*-mer threshold that corresponds to the user-defined NW distance threshold (e.g., 3% dissimilarity of 16S sequences). In the *initial clustering* step, CLUSTOM calculates *k*-mer distance of each pair in the 16S input sequences, chooses sequence pairs below the *k*-mer threshold, connects the two sequences of the individual pairs to each other and constructs an initial network only with the selected sequence pairs [11]. CLUSTOM then searches for the sequence to which the largest number of other sequences is directly connected. This highly connected node in the network is then treated as *seed* that, along with its directly connected sequences, is used to build an initial cluster. This process is repeated to determine additional seeds and clusters from the remaining sequences until each cluster is left with only one sequence (i.e., a singleton). The *refinement* step then calculates NW distance for each pair of *seed* sequences as well as the singletons determined in the *initial clustering* step and builds a refined network. The sequence to which the largest number of sequences is connected is treated as the *refined seed*. CLUSTOM then determines the first final cluster consisting of the *refined seed*, sequences in the refined network directly connected to the *refined seed* (neighbors) and sequences in the initial network directly connected to either the *refined seed* or its neighbors [11]. Next, final clusters are iteratively determined in the same manner as described in the *initial clustering* step. Although CLUSTOM-CLOUD clusters sequences in a similar way, the workflow is different because of distributed processing based on IMDG and additional enhancements. Below, we outline the workflow of CLUSTOM-CLOUD focusing primarily on the upgrades that led to significant performance improvement in sequence clustering (Fig 2). More methodological details and rationale for the CLUSTOM workflow can be found in [11].

### Preprocessing

**Fig 3:**
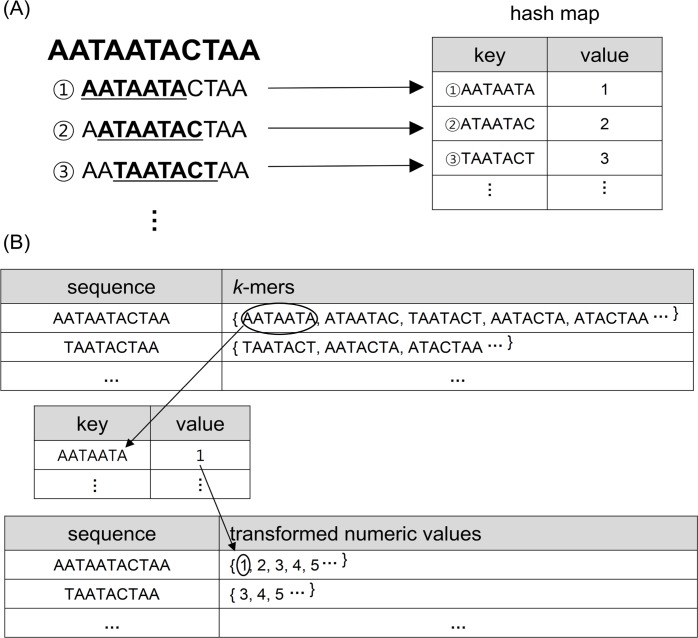
Representation of *k*-mer transformation method. The diagram summarizes the layout of the *k*-mer transformation method. (A) All *k*-mer strings in the input sequence dataset along with non-redundant numeric values are loaded into hash map. (B) All *k*-mer in each sequence are replaced with numeric values corresponding to each key in hash map.

Before loading data into IMDG, each input FASTA file consisting of 16S rRNA reads is preprocessed by, (i) the removal of duplicate sequences and (ii) transformation of *k*-mer strings into numeric values (Fig 2). Sequence data produced by microbial diversity projects may contain duplicate sequences depending on the properties of microbial diversity in samples [21]. In CLUSTOM, duplicates were used to find the most connected nodes during the *initial clustering* and *refinement* steps. However, they were retained through the entire clustering procedure and unnecessarily consumed additional computing time and resources. Therefore, an additional feature of CLUSTOM-CLOUD is the dynamic treatment of duplicate reads. Specifically, duplicates are removed before loading data into IMDG but are recovered during the clustering steps. Similar to other clustering programs (e.g., mothur, ESPRIT-Tree, UCLUST, Swarm, etc.), the dereplication contributes to reducing the overall processing time. Similarly, calculation of *k*-mer distances between each pair of input sequences was the most time-consuming step in CLUSTOM, requiring polynomial complexity *O(N*^*2*^*)*, with *N* representing the total number of sequences. Therefore, performance could be significantly improved if *k*-mer distance calculation time was somehow accelerated. We accomplished this task by loading all *k*-mer strings and their corresponding number of occurrences directly into memory before distance calculation. Because this operation still involved a huge number of comparisons between *k*-mer strings, we transformed *k*-mers into numeric values using the hash map data structure (Fig 3A). Specifically, each *k*-mer in sequence (key) was transformed into a non-redundant numeric value corresponding to each key in the hash map (Fig 3B). For *k*-mer strings not present in the hash map, a new *key* and *value* was dynamically created.

### Initial clustering

The *k*-mer distance between each input sequence pair is calculated using parallel and distributed computing (Fig 2A). All tasks are distributed to *Cluster Nodes* sequentially by the *Job Tracker* and then processed in parallel using multi-threads by *Task Tracker*. To reduce communication overhead associated with sending data between nodes and IMDG, intermediate results generated during the process of the unit task are first saved in the local memory of the processing node. Upon completion of the unit task, processed results are merged sequentially into IMDG (Fig 2B, Merge phase) with simultaneous removal of intermediate data from the local memory. The workflow is similar to that of Hadoop MapReduce [22] but differs in the timing of when processed results are merged. In MapReduce, results are merged upon completion of all distributed tasks. In turn, CLUSTOM-CLOUD merges processed results after each task is finished to prevent shortage of memory in local nodes. Once all tasks are completed and results are merged, a set of representative initial clusters is determined (Fig 2C) based on *k*-mer distance calculations and *seed* selection. Clusters are chosen via parallel processing on the *Application* node because cluster-determination via distributed processing can be relatively less efficient due to higher communication cost and load imbalance. However, distributed processing functionality is also supported as an alternative in CLUSTOM-CLOUD.

### Refinement

For each pair of the most connected sequences in the *initial clustering* step, NW distances are calculated as described above (Fig 2D and 2E). Once all distributed tasks are finished, final clusters are determined, similar to CLUSTOM [11] (see also Fig 2F).

### Output

Results are archived to the local file system of *Application*. Three output files identical in format and structure to CLUSTOM are generated from the final cluster analysis (Fig 2): (i) sequences per OTU (FASTA), (ii) representative sequences of OTUs (FASTA), and (iii) number of sequences per OTU (Plain text). All data are cleared from IMDG following output generation.

### Dataset preparation for evaluation of running time

The composition of microbial populations largely depends on the features of the natural environment [23]. Some habitats favor the growth of certain species while restrict the others. Therefore, sequence data extracted from different environmental samples exhibit varying levels of microbial complexity and this property naturally relates to the performance of clustering algorithms. Therefore, we prepared test datasets with varying levels of microbial complexity to assess the running time of CLUSTOM-CLOUD. We first retrieved 16S rRNA sequences corresponding to 18 different human body sites from the data archive of the Human Microbiome Project (http://hmpdacc.org/HM16STR, download date: May 14, 2015). Microbiome sequences were generated using the Roche-454 FLX Titanium platform (hereafter 454-HMP) and were already trimmed and error-corrected. Additionally, we filtered out sequences below 300 bp from each of the 18 sampled locations. A set of 10 K sequences was then randomly extracted from each sampled location and was analyzed by CLUSTOM for richness and evenness, two well-known indicators of microbial diversity [3, 4]. Based on the alpha-diversity patterns, body sites were pooled into three datasets exhibiting *high* (2,666,826 total reads containing ~19.67% duplicates), *intermediate* (2,520,233 reads containing~25.77% duplicates), and *low* (1,234,923 reads containing ~36.73% duplicates) microbial complexity (S1 Fig). From each pooled dataset, we randomly extracted 50 K, 100 K, 150 K, and 200 K sequences (average sequence length 474–482 bp with a standard deviation of 55–57) for performance analysis. Additionally, we randomly sampled one million reads (~23.58% duplicates, average length = 478 bp, standard deviation = 56) from the three datasets to evaluate the running time of CLUSTOM-CLOUD under the Amazon EC2 cloud-computing environment.

### Test environment

The running time evaluation was conducted on a small computer cluster consisting of one *Application* and 10 *Cluster Nodes* using the three datasets described above (*high*, *intermediate*, and *low*). Each node was equipped with a single quad-core Intel(R) Xeon(R) CPU E3-1270 v3 @ 3.50GHz, 32 G of ECC RAM, 2 TB hard disk (40 total cores, 320 GB total memory), and a Cent OS 6.4 operating system connected to 1 Gigabit Ethernet. In addition, Amazon EC2 clusters of one *Application* and 20, 30, and 40 *Cluster Nodes* were tested under the cloud-computing environment using one million reads. In each case, *Application* was a High-CPU Extra Large Instance (virtualized 64-bit computer with 60 GB of memory) equivalent to 32 processor cores clocked at approximately 2.8 GHz, whereas each *Cluster Node* was an EC2 High-Memory Extra Large Instance (virtualized 64-bit computer with 30.5 GB of memory) equivalent to four processor cores clocked at approximately 2.5 GHz.

### Determination of optimal granularity

The amount of work assigned to parallel and distributed tasks (granularity) is the major factor influencing the performance of parallel and distributed computing systems [24]. If granularity is too fine (small size), communication overhead between tasks may take longer than computation, resulting in performance degradation. In contrast, coarse granularity (large size) implies improved performance but may cause load imbalance due to unequal allocation of workload, leading to some nodes or processors being idle [25]. To determinate the optimal granularity of CLUSTOM-CLOUD, we measured the computational time required for calculating *k*-mer (*initial clustering*) and NW (*refinement*) distances for a 100 K sample of *high* complexity by repeatedly changing the number of sequence pairs distributed to each *Cluster Node* (1 K, 1.5 K, 2 K, 2.5 K and 3 K) and the total number of nodes (5 and 10) under the test environment described above (i.e., small computer cluster connected to 1 Gigabit Ethernet). Although granularity could also fluctuate somewhat based on the network environment, we executed tests at 1 Gigabit network speed, which is the recommended and default speed to execute CLUSTOM-CLOUD. Moreover, users can customize the number of sequence pairs distributed to each task. Therefore, optimal granularity calculated in the test environment is expected to be a close approximation of the real environment.

### Fine-grained task distribution

**Fig 4:**
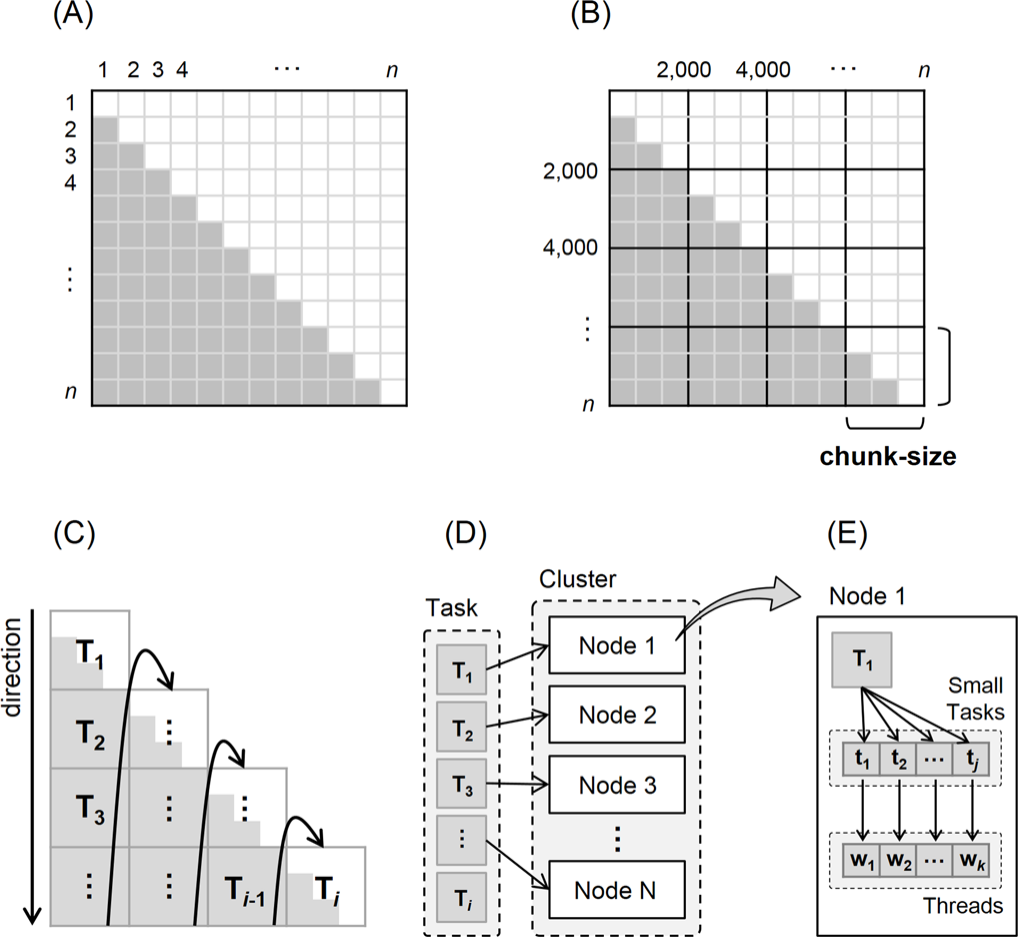
Fine-grained task distribution in CLUSTOM-CLOUD. The figure summarizes the workflow of distributed processing in CLUSTOM-CLOUD. (A) The number of all possible sequence pairs that need to be compared for distance calculation is represented as a right-angled triangle; *n* represents the total number of sequences. (B) A chunk-size based on system granularity is determined to distribute only a fixed number of sequence pairs (shown here with 2 K) to each cluster node. (C) Each task (e.g., T_*i*_) is assigned to nodes from top to bottom and left to right. (D) Each node takes and processes tasks in the order of task priority. (E) The assigned task (T_*i*_) is divided into smaller sub-tasks (t_*j*_) and processed in parallel using multi-threads (w_k_) depending on the number of threads on the cluster node.

Calculation of *k*-mer and NW distances between every sequence pair was the major contributing factor to computational time in CLUSTOM. Because all distributed tasks were processed independently in each node, performance could suffer from load imbalance. To overcome these limitations, we developed a fine-grained task distribution method in CLUSTOM-CLOUD (Fig 4): (i) for *n* sequences, the total number of sequence pairs to be compared are given by the relation n*(n-1)/2, shown as the right-angled triangle in Fig 4A; (ii) a fixed number of sequence pairs are assigned to each task based on the optimal granularity of the system (e.g., fixed-size chunks of 2 K sequence pairs shown in Fig 4B); (iii) tasks are evenly distributed to cluster nodes by *Job Tracker* in the order of top to bottom and left to right (Fig 4C and 4D); and (iv) then processed in parallel using multi-threading by *Task Tracker* in Fig 4E. The task (T_*i*_) assigned by *Job Tracker* of *Application* will be divided into smaller sub-tasks (t_*j*_) by *Task Tracker* on each cluster node. The sub-tasks (t_*j*_) will be processed in parallel way using multi-threads (w_*k*_) in compliance with the number of threads of each cluster node. Table 1 shows the execution time of the cluster node.

**Table 1:** CLUSTOM-CLOUD running time for each step according to the complexity of the microbial diversity^a^.

		*Initial clustering*		*Refinement*		
Dataset	Size	*k*-mer distance calculation^b^	Cluster determination	NW calculation	Cluster determination	Total
***High***	50 K	8.6 m	0.2 m	25.1 m	0.6 m	34.5 m
	100 K	30.2 m	1 m	26 m	1.4 m	58.6 m
	150 K	68.2 m	2.6 m	45.9 m	2.6 m	119.3 m
	200 K	111.8 m	7.1 m	56.2 m	2.7 m	177.8 m
***Intermediate***	50 K	8.2 m	0.2 m	19.7 m	0.6 m	28.7 m
	100 K	26.2 m	0.8 m	22.5 m	1.4 m	50.9 m
	150 K	55.3 m	2.4 m	24.9 m	1.9 m	84.5 m
	200 K	96.8 m	5.6 m	24.5 m	2.8 m	129.7 m
***Low***	50 K	6.3 m	0.3 m	7.1 m	0.6 m	14.3 m
	100 K	19.4 m	1.4 m	16.9 m	1.2 m	38.9 m
	150 K	41.1 m	4.2 m	18.5 m	1.8 m	65.6 m
	200 K	68 m	9.1 m	20.2 m	2.4 m	99.7 m

^a^ measured using one application node and 10 cluster nodes, each equipped with a single quad-core Intel(R) Xeon(R) CPU E3-1270 v3 @ 3.50GHz and 32 GB of RAM.

^b^ This includes running time required at the steps of dereplication and *k-mer threshold determination*.

### Evaluation of *k*-mer transformation approach

To evaluate whether implementation of *k*-mer transformation improved clustering efficiency in terms of running time and memory usage, we analyzed 100K sequence datasets (*high*-, *intermediate*- and *low*-complexity) using CLUSTOM-CLOUD *with*- and *without k-mer transformation method*. In this exercise, we avoided sequence dereplication that significantly influences the *k*-mer distance calculation time. Tests were conducted three times for each dataset on the small computer cluster in the lab.

### Speed performance evaluation in the small computer cluster in the lab

To evaluate the speed of CLUSTOM-CLOUD in clustering sequence datasets exhibiting *high*, *intermediate*, and *low* microbial complexity, we measured the running time and memory usage for analyzing 50 K, 100 K, 150 K, and 200 K samples by repeating three times per each dataset. All experiments were conducted using the small computer cluster in the lab. Since running time also depends on genetic distance thresholds and other program options, we used 3% dissimilarity threshold, which is regarded as standard to resolve species [7, 26], and other default options. Even though a global threshold may not appropriately and accurately delimit different OTUs, the 3% dissimilarity of 16S sequences is the most conventional threshold in the fields of molecular-sequence based microbial ecology. So, the adoption of a single threshold is not problematic in evaluating the performance of CLUSTOM-CLOUD under the given computing environment.

### Speed performance evaluation under the cloud-computing environment

To evaluate the speed performance and scalability of CLUSTOM-CLOUD in analyzing large amount of sequence data, we measured the running time for processing one million reads using Amazon EC2 clusters consisting of one *Application* unit and 20, 30, and 40 *Cluster Nodes*. All tests were performed under default options and a distance threshold of 3%.

### Running time comparison to CLUSTOM

CLUSTOM was optimized for parallel processing in a single node. In turn, CLUSTOM-CLOUD is designed to operate under distributed computing environment based on IMDG data structure. Therefore, performance of CLUSTOM-CLOUD deteriorated in a single node likely caused by network overhead to connect IMDG. To provide a realistic assessment of CLUSTOM-CLOUD performance relative to CLUSTOM, we developed a new Java-based version of CLUSTOM (CLUSTOM-J) optimized for parallel processing in a single node and identical to the implementation of CLUSTOM-CLOUD except that it did not use the IMDG data structure. To evaluate clustering performance between CLUSTOM-J and CLUSTOM, we measured the running time for analyzing 100 K sequences extracted from each of the three datasets (*high*, *intermediate*, and *low*) using the single node under default options and distance threshold of 3%. Since CLUSTOM can only be executed under Linux environment installed with GCC 4.1.2 or an earlier version, we prepared a single computing node equipped with 64-core AMD Opteron 2.3GHz processor and 256 GB of ECC RAM.

### Implementation

CLUSTOM-CLOUD is implemented in Java (ver. 1.7). Hazelcast (ver. 3.3.5) (http://hazelcast.com) was used to implement the distributed processing system based on IMDG and to support platform independence and various computing environments. The program is composed of two executables for IMDG: *Cluster* and *Application*. Input to CLUSTOM-CLOUD is a FASTA file of 16S rRNA sequences and an XML configuration file (see S1 Text) that includes the IP addresses of *Cluster Nodes*, data size for allocation, and number of threads per node. All the details of computing system requirements, installation and configuration that are necessary for running CLUSTOM-CLOUD can be found in S2 Text. Output is three files, similar to CLUSTOM: (i) sequences per OTU (FASTA), (ii) the representative sequences of OTUs (FASTA), and (iii) the number of sequences per OTU (plain text). CLUSTOM-CLOUD and its user guide are freely available at http://clustomcloud.kopri.re.kr.

### Clustering accuracy evaluation

In order to compare the clustering accuracy of existing programs against CLUSTOM-CLOUD, we prepared a mock community sequence dataset. We first retrieved 16S rRNA pyrosequences of a mock community that was constructed by pooled DNA of 21 human-associated prokaryotic strains with even concentration (SRR072219 download on December 28, 2015). Tags and low-quality reads were trimmed out using PyroTrimmer [2] prior to sequence clustering. The V1-3 region of sequences was extracted using an in-house script. Next, individual sequences were BLASTn-searched against the dataset of [27] containing 16S sequences of the 21 strains. Taxonomic information of the top BLAST hits was then assigned to the corresponding query sequences. From 35,106 sequences with taxonomic information, 10 K sequences (hereafter HMP-Mock-community, average length = 439 bp, standard deviation = 46) were randomly extracted and clustered using CLUSTOM-CLOUD, CLUSTOM, DOTUR-AL-PSA (AL = average-linkage; PSA = pairwise sequence alignment), ESPRIT-Tree, mothur-AL-MSA (MSA = multiple sequence alignment), mothur-AL-PSA, UCLUST and Swarm. In case of Swarm, two different distance thresholds (*d* = 1 and 3) were used in clustering while the remaining programs were ran using 3% (species-like) and 5% (genus-like) conventional thresholds. Specifically, UPARSE that discards singletons by default may result in the elimination of rare OTUs followed by distortion of natural microbial community [28, 29]. Since low abundance taxa sometimes play important roles in certain environments, they should be retained in evaluating the clustering accuracy of programs. Therefore, we used UCLUST in this comparative exercise that is the previous version of UPARSE and still produces singletons in its clustering results. We then evaluated the clustering accuracy of individual programs by counting how many pairs of sequences were correctly clustered. Each of the possible sequence pairs were assigned to one of the four different status categories: true positive (TP), false positive (FP), true negative (TN) and false negative (FN). Sequence pairs derived from the same strain and clustered together in a single OTU were regarded as TP. Sequence pairs clustered in an OTU but originated from different strains were regarded FPs. Sequence pairs originated from the same strain but belonging to different OTUs were FNs. While, the remaining sequence pairs were regarded as TNs. We then calculated precision TP/ (TP+FP) and recall TP/ (TP+FN). Consequently, precision increased when the OTUs included greater number of sequences derived from same strain while the recall decreased when the sequences from a single strain were more evenly distributed in different OTUs. The harmonic mean of the precision and recall was computed using formula of F-measure II (*F*_*2*_) [30].

## Results

### Determination of optimal granularity

Determining optimal granularity is critical for the success of each algorithm in parallel and distributed computing. Here, granularity refers to the optimal number of sequence pairs to be compared by each *Cluster Node* and was determined by repeatedly assigning 1 K, 1.5 K, 2 K, 2.5 K, and 3 K sequence pairs from 100 K *high*-complexity dataset to 5 and 10 nodes (S2 Fig). The computational time required for calculation of *k*-mer and NW distances was then compared to determine the optimal number of sequence pairs and nodes required for maximum performance. The experiment revealed that distance calculation for 2 K sequence pairs required minimum processing time in either 5 (~5,500 seconds) or 10 nodes (3,300 seconds). In turn, calculation time increased considerably when the number of sequence pairs was reduced below 2 K (~7,300 and 5,900 seconds for 5 nodes and ~3,500 and 3,400 seconds for 10 nodes), likely caused by communication overhead. Similar patterns were observed when the number of sequence pairs was increased to either 2.5 K (6,160 seconds in 5 nodes and 3,660 for 10) or 3 K (5,700 and 4,350), resulting in extra running time likely caused by low computation-to-communication ratio. In other words, merging the processed results for a larger number of sequence pairs after the completion of each task required more communication time than merging results from smaller datasets. Therefore, we selected 2 K sequence pairs as the optimal granularity in the distributed task of CLUSTOM-CLOUD, which is set as the default option but can be customized by users.

### *k*-mer transformation utilized less memory and enabled faster execution

**Fig 5:**
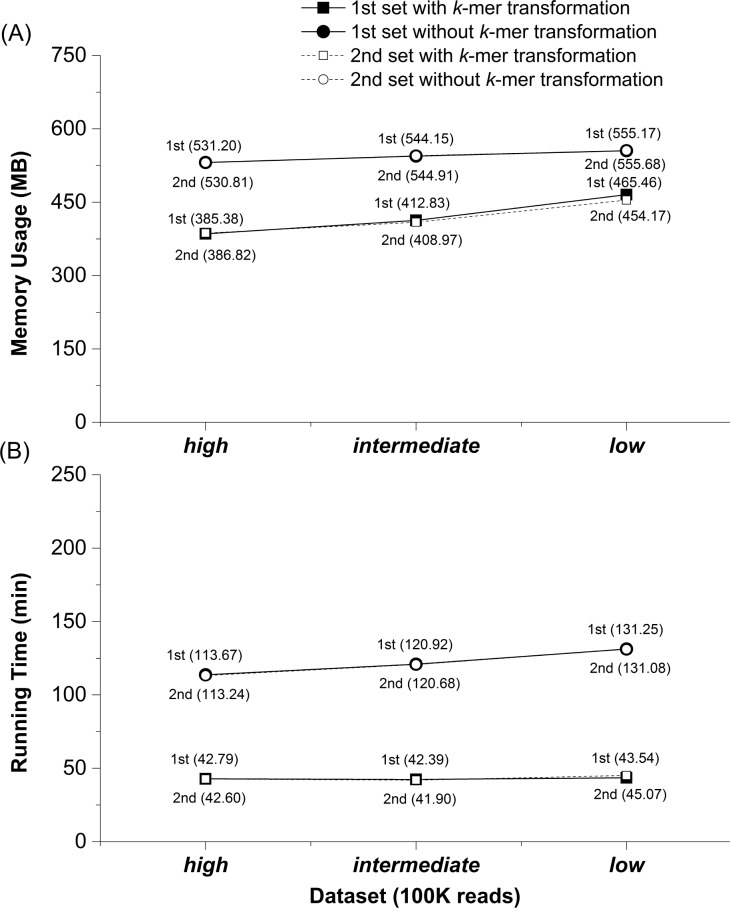
Running time and memory usage evaluation of the *k*-mer transformation method. Comparison of the memory usage (A) and running time (B) were performed with and without *k*-mer transformation method only at the *k*-mer distance calculation step. Two of 100K 16S sequences were independently and randomly extracted from the sequence datasets of *high*-, *intermediate*- and *low*-complexity. For each of the six different sequence datasets, the running time and memory usage were measured three times independently.

We assessed the running time (minutes) and memory usage (MB) by processing 100 K sequences in consideration of both cases of *with*- and *without the k-mer transformation method* (Fig 5). The 100 K datasets of *high*-, *intermediate*- and *low*-complexity were prepared from the 454-HMP data. This sampling was repeated twice independently. Each of the six datasets was analyzed three times. For this exercise, sequence dereplication was not conducted because it significantly influenced the *k*-mer distance calculation time. The exercise revealed that using the *k*-mer transformation required approximately 42.8 (42.6 in the second sampling), 42.4 (41.9), and 43.5 (45.1) minutes of running time (Fig 5B) and approximately 385 (387), 413 (409), and 465 (454) MB of memory in decreasing order of dataset complexity (Fig 5A). In turn, the running time without implementing the *k*-mer transformation was approximately 113.7 (113.2), 120.9 (120.7), and 131.3 (131.1) minutes respectively (Fig 5B), and memory usage was approximately 531.2 (530.8), 544.2 (544.9), and 555.2 (555.7) MB, respectively (Fig 5A). Therefore, *k*-mer transformation significantly decreased both running time (by at least two-fold) and memory usage (down by 16–27%). Importantly, *k*-mer transformation was largely neutral to dataset complexity, as relatively similar time and memory storage were required for processing data from each of the three complexity datasets. Strictly, the result revealed that decreasing data complexity required slightly more memory and running time (Fig 5A and 5B). In fact, this is not a surprising trend in both CLUSTOM and CLUSTOM-CLOUD. The sequence datasets of the *low* complexity include larger number of nearly identical 16S sequences than the datasets of either *intermediate* or *high* complexities. Consequently, increasing the number of highly similar sequences results in increasing the number of sequence pairs that satisfy the *k*-mer distance threshold (e.g., conventionally 3% dissimilarity of 16S sequences). Since the selected sequence pairs should be loaded to the RAM, the *low* complexity data requires more memory and running time than the other datasets. In short, *k*-mer transformation feature is a useful add-on in CLUSTOM-CLOUD that enables at least two-fold decrease in processing time while utilizing significantly less memory.

### Clustering speed performance is indifferent to the nature of dataset

**Fig 6:**
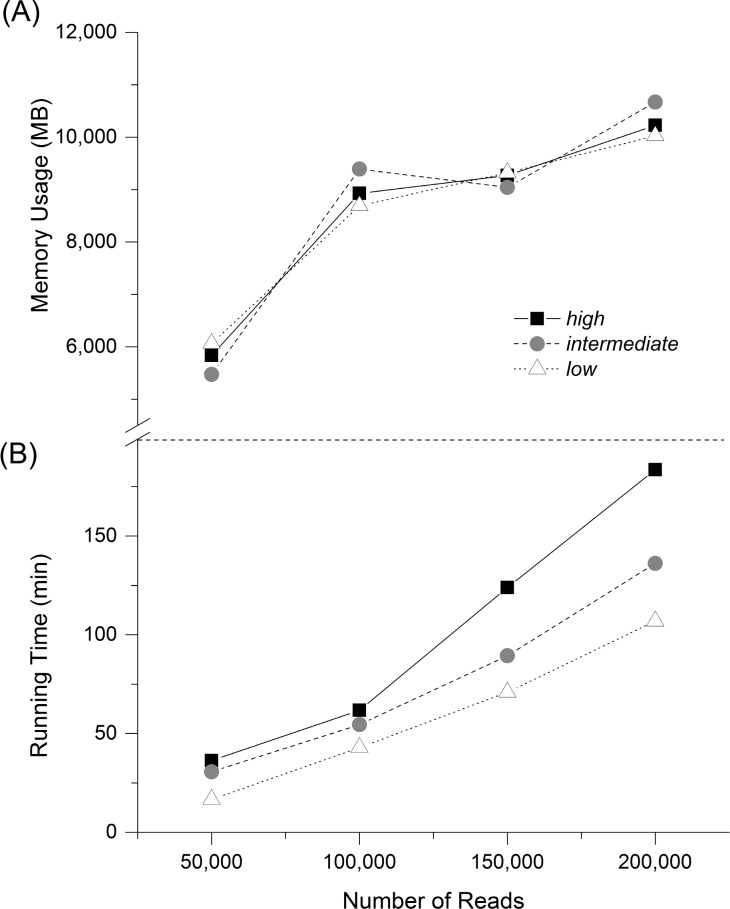
Running time of the whole process according to the complexity of the microbial diversity. Running time (A) and memory usage (B) of CLUSTOM-CLOUD were measured by analyzing 50 K, 100 K, 150 K, and 200 K sequences in *high*-, *intermediate*-, and *low*-complexity datasets (3% distance threshold). The measures were repeated three times per dataset and the average values are plotted.

We compared the performance of CLUSTOM-CLOUD in processing 50 K, 100 K, 150 K, and 200 K reads randomly extracted from the *high*-, *intermediate*-, and *low*-complexity datasets. The analysis revealed that running time was primarily determined by the size of the input dataset (as expected) and was secondary to sample’s complexity (Fig 6). For example, processing time for smaller datasets (e.g., 50 K and 100 K) from each of the three complexity classes did not differ significantly. However, running time gap widened significantly when the number of input reads was increased to either 150 K or 200 K (Fig 6B). Because high-complexity datasets possess fewer duplicates, additional time is required to calculate *k*-mer and NW distances, resulting in extra processing time (Table 1). In turn, memory usage was largely indifferent to the complexity of microbial diversity and was only dependent on the number of input sequence reads (Fig 6A). Because CLUSTOM-CLOUD allocates a certain amount of total memory in advance to improve performance, memory usage was nearly consistent at ~10 GB per node for 100 K, 150 K, and 200 K samples (Fig 6A).

### Cloud-computing performance in Amazon EC2

To evaluate the performance and scalability of CLUSTOM-CLOUD, we performed separate experiments using 20, 30, and 40 EC2 High-Memory Extra Large Instance *Cluster Nodes* (plus one High-CPU Extra Large Instance node of *Application*) for one million sequences. Table 2 shows the wall-clock running time for each experiment. Remarkably, CLUSTOM-CLOUD is capable of clustering one million reads in less than 12 hours of wall-clock time using 40 *Cluster Nodes* despite the polynomial complexity *O(N*^*2*^*)* of the *k*-mer distance calculation step. The running time of CLUSTOM-CLOUD on 160 cores of the EC2 cluster was approximately two-fold faster than 80 cores of the EC2 cluster, indicating high scalability with the increasing number of cores. Typically, distributed and parallel computing systems exhibit sub-linear growth in speed with increasing numbers of cores. Although CLUSTOM-CLOUD shows a similar sub-linear performance growth, the deviation is very slight. It implies that CLUSTOM-CLOUD is relatively more effective for distributed and parallel processing. To summarize, CLUSTOM-CLOUD can rapidly and stably cluster a large number of sequences under the cloud-computing environment.

**Table 2:** Time and cost of running one million reads on CLUSTOM-CLOUD.

EC2 nodes	1 app^a^, 20 clusters^b^	1 app^a^, 30 clusters^b^	1 app^a^, 40 clusters^b^
**Processor cores**	80	120	160
**Wall clock time**	20 h:38 m	14 h:05 m	11 h:34 m
**Cluster setup time**	5 m	5 m	5 m
**Reads uploading time**	36 m	36 m	36 m
***k*-mer distance calculation time**	16 h:34 m	11 h:03 m	8 h:50 m
**Initial cluster determination time**	55 m	27 m	25 m
**NW distance calculation time**	2 h:11 m	1 h:38 m	1 h:22 m
**Final cluster determination time**	17 m	16 m	16 m

^a^ specification of an application node in Amazon EC2 is c3.8xlarge.

^b^ specification of a cluster node in Amazon EC2 is c4.2xlarge.

### CLUSTOM-CLOUD can be up to five times faster than CLUSTOM

We compared the running time (minutes) difference between CLUSTOM and CLUSTOM-J for analyzing 100 K sequences extracted from *high*-, *intermediate*-, and low-complexity datasets in a single node. CLUSTOM-J using 20 CPU cores required ~207, 120, and 79 minutes for *high*, *intermediate*, and *low* datasets respectively. In turn, CLUSTOM under similar conditions required ~ 237, 378, and 407 minutes respectively. For all experiments, CLUSTOM-J was much faster than CLUSTOM despite the difference in programming language (Java versus C). Remarkably, CLUSTOM-J was about five times faster than CLUSTOM in processing the *low* complexity dataset. The experiment indicated that implementation of IMDG and its additional enhancements in CLUSTOM-CLOUD significantly improved the overall clustering performance of CLUSTOM.

### Comparative exercise on the clustering accuracy

**Fig 7:**
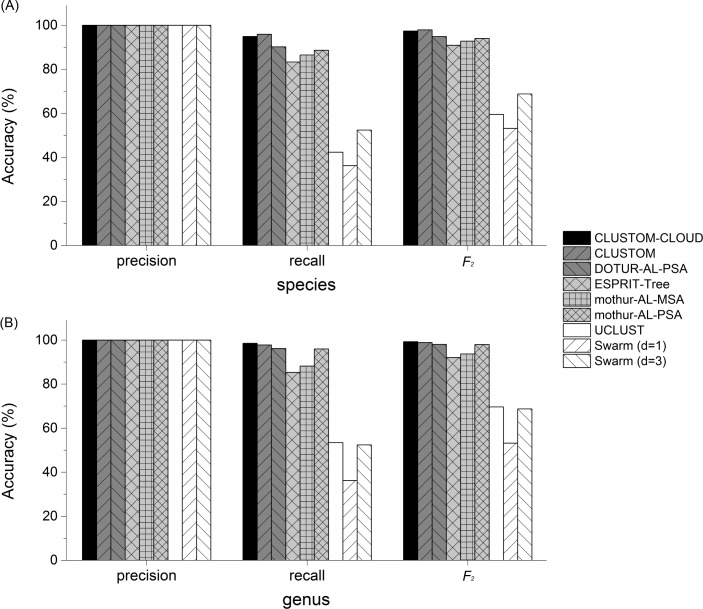
Comparative accuracy test of existing clustering programs. The clustering accuracy of CLUSTOM, CLUSTOM-CLOUD, DOTUR-AL-PSA, ESPRIT-Tree, mothur-AL-PSA, mothur-AL-MSA, UCLUST and Swarm was performed based on 16S rRNA pyrosequences of a mock community that was constructed by pooled DNA of 21 human-associated prokaryotic strains with even concentration (HMP-Mock-community). The precision and recall metrics as well as their *F*_*2*_ values were used to compare the clustering accuracy of the eight programs at the species (A) and genus (B) levels.

Comparative analysis using HMP-Mock-community was performed to evaluate the clustering accuracy of CLUSTOM-CLOUD with other programs. We ran CLUSTOM, CLUSTOM-CLOUD, DOTUR-AL-PSA, ESPRIT-Tree, mothur-AL-PSA, mothur-AL-MSA and UCLUST using 3% and 5% conventional distance thresholds that likely correspond to the taxonomic levels of species and genus, respectively. As mentioned in Methods, we used distance value (*d*) of 1 and 3 to cluster sequences using Swarm. The clustering results revealed that the number of OTUs determined by individual programs varied. For example, CLUSTOM-CLOUD produced 97 and 49 OTUs at 3% and 5%, respectively. Other programs produces OTUs as follows: CLUSTOM (65 and 30 at 3% and 5%, respectively), DOTUR-AL-PSA (125 and 52 at 3% and 5%), ESPRIT-Tree (300 and 145 at 3% and 5%), mothur-AL-MSA (356 and 217 at 3% and 5%), mothur-AL-PSA (150 and 60 at 3% and 5%) and UCLUST (454 and 226 at 3% and 5%). The number of OTUs determined by Swarm with *d* = 1 (3,579) was larger than 2,473 OTUs with *d* = 3. Consequently, the average-linkage-based clustering algorithms (CLUSTOM, CLUSTOM-CLOUD, DOTUR-AL-PSA, mothur-AL-PSA and mothur-AL-MSA) produced smaller number of OTUs than UCLUST (greedy-heuristic) and Swarm (single-linkage hierarchical clustering). The accuracy test revealed that the precision values of all the programs reached 100% nearly or completely (Fig 7). On the other hand, the recall accuracy of the programs largely varied. For example, the recall values of Swarm (36% at *d* = 1 and 52% at *d* = 3, respectively) and UCLUST (42% and 53% at 3% and 5%, respectively) were significant lower than those of other programs. The recall values in details are as follows: CLUSTOM-CLOUD (95% and 99% at 3% and 5%), CLUSTOM (96% and 98% at 3% and 5%, respectively), DOTUR-AL-PSA (90% and 96% at 3% and 5%), ESPRIT-Tree (83% and 85% at 3% and 5%), mothur-AL-MSA (86% and 88% at 3% and 5%) and mothur-AL-PSA (89% and 96% at 3% and 5%). Consequently, the harmonic means, *F*_*2*_ values, show that both CLUSTOM-CLOUD and CLUSTOM are accurate relative to the other average-linkage based hierarchical clustering programs and significantly more accurate than greedy-heuristic clustering (UCLUST) and single-linkage hierarchical clustering (Swarm) programs.

## Discussion

We present CLUSTOM-CLOUD, a distributed clustering program that can efficiently and accurately cluster 16S sequences under distributed and cloud-computing environments. CLUSTOM-CLOUD is a significant upgrade to its predecessor, CLUSTOM. The enhancements include: (i) implementation of *k*-mer transformation, (ii) removal of duplicate sequences (dereplication), and importantly (iii) the implementation of IMDG technology to store data directly into RAM rather than hard disks of individual nodes. Importantly, CLUSTOM-CLOUD inherits the high accuracy of its ancestor CLUSTOM, as also confirmed by the comparative exercise (Fig 7). The *k*-mer transformation helps to reduce the *k*-mer distance calculation time during *initial clustering*. Since all *k*-mer strings are transformed into small-byte numerical values using hash map, this implementation significantly reduced both the running time (twice as fast) and memory utilization (down by 16–27%) relative to implementation without *k*-mer transformation (Fig 5). Consequently, *k*-mer transformation method is a useful add-on to calculate *k*-mer distances for datasets. In turn, duplicates are important to determine the initial and final clusters but are not required during other steps of processing. Therefore, CLUSTOM-CLOUD removes duplicates before loading data into *Cluster Nodes* and recovers them later for cluster determination. In contrast, duplicates were part of the entire clustering process in CLUSTOM, costing more computational time and resources. Both features confer ~5 times faster execution in CLUSTOM-CLOUD relative to CLUSTOM. Finally, compared to other commonly used existing distributed computing technologies such as MPI and MapReduce of Hadoop, IMDG provides parallel and distributed computing using in-memory storage to allow rapid processing of sequence datasets and relatively convenient execution. By using IMDG technology, CLUSTOM-CLOUD supports various computing environments including cloud computing with relatively easier installation and configuration steps than the other distributed and cloud-computing programs. These considerations identify the IMDG-based distributed computing architecture of CLUSTOM-CLOUD as a better alternative to either MPI or MapReduce of Hadoop.

To assess the running time of CLUSTOM-CLOUD, we analyzed randomly sampled 454-HMP datasets of various sizes exhibiting *high*, *intermediate*, and *low* microbial complexity. Processing 200 K reads from each dataset required only between 100 and 178 minutes when ran on a small laboratory computer cluster with 10 nodes (Table 1). This indicates that CLUSTOM-CLOUD is still useful to cluster hundreds of thousands of 16S sequences without the need for expensive high-performance machines, although greedy-heuristic or single-linkage based programs such as UCLUST and Swarm require significantly less computing resources. To demonstrate the scalability of CLUSTOM-CLOUD, we analyzed one million sequences using 20, 30, and 40 nodes on Amazon EC2 that required ~20, 14, and 11 hours, respectively (Table 2). This represents how CLUSTOM-CLOUD shows substantial improvement in running time by the addition of computing nodes indicating high scalability. Moreover, millions of sequences can be analyzed under the cloud-computing environment (e.g., Amazon EC2) supported by CLUSTOM-CLOUD.

Different factors influence the speed performance of CLUSTOM-CLOUD. As expected, the amount of input data is directly related to running time (Fig 6B). Running time also increases for datasets exhibiting high complexity, especially for large datasets (Fig 6). This is largely due to the presence of fewer duplicates in high-complexity datasets [21] that require additional computation time in calculating *k*-mer and NW distances. For example, running time for small datasets (below 100 K) was not significantly different. However, for larger datasets (e.g., 150 K and 200 K), processing time for the *low*-complexity dataset was ~1.3–1.8 times faster than for the *intermediate* and *high* datasets. Since IMDG allows us to rapidly access and process data in the shared memory pool, overall performance is greatly affected by the network environment. A network with high stability and over 1 Gigabit speed is therefore recommended for superior performance. Finally, parallel processing in the *Application* node is used as default to determine the initial and final clusters. Therefore, equipping the *Application* node with extra cores will directly increase performance.

The relationship between accuracy and computational complexity is a trade-off in all algorithms and CLUSTOM-CLOUD is no different. Although CUSTOM-CLOUD significantly reduces memory usage and running time of CLUSTOM through dereplication and *k*-mer transformation methods, it is slower than greedy heuristic based clustering algorithms (actual needs of computing resources and speed) because its computational complexity is quadratic. However, as shown in Fig 7, the average-linkage based hierarchical clustering methods show accurate results compared to the greedy heuristic clustering algorithm such as UCLUST. Among clustering methods, CLUSTOM and CLUSTOM-CLOUD exhibited the highest accuracy rate than any other program. Swarm that is based on single-linkage hierarchical clustering achieves clustering accuracy similar to UCLUST. In fact, the previous study of Swarm showed that it performed robustly regardless of the value *d* [12]. Therefore, it is hard to expect that the accuracy of Swarm increases by adjusting the *d* values. Although applying a breaking phase helps Swarm delimit the natural limit of OTUs, it still seems to suffer from the chain reaction problem of the single-linkage hierarchical clustering methods. However, the result of the comparative accuracy test may differ depending on alignment quality, distance calculation method, sequencing filtering, the selected region of 16S sequence, sequencing platforms, clustering distance thresholds and metrics for measuring the accuracy. Therefore, it is further necessary to conduct more comprehensive and precise experiments on the evaluation of the clustering algorithms.

In conclusion, CLUSTOM-CLOUD is limited to relatively smaller datasets and is slower than ultrafast sequence clustering programs such as UCLUST and Swarm. However, it outperforms several other accurate clustering alternatives such as CLUSTOM, DOTUR-AL-PSA, mothur-AL-PSA and mothur-AL-MSA in terms of computational speed and handling of relatively larger datasets. Therefore, CLUSTOM-CLOUD is a good alternative to rapidly and accurately cluster large 16 rRNA sequences that cannot be handled by several other accurate clustering programs. It also becomes the ideal algorithm to implement when the laboratory has the infrastructure of many small computers that can be better utilized as distributed computing system. Although RAM is more expensive than hard disk, its cost is consistently declining. Therefore, in-memory computing technology such as IMDG provides a promising and reliable solution for the rapid analysis of biological data. To our knowledge, the present study is the first application of IMDG in bioinformatics. Its successful integration into CLUSTOM resulted in significant performance improvement and speedier execution. Evolution of CLUSTOM into CLUSTOM-CLOUD potentially serves as a starting point to consider the broader use of IMDG-based technologies in life sciences.

## Supporting Information

S1 Fig Classification of human microbiome datasets according to microbial complexity. 16S rRNA sequence data from 18 human body sites was analyzed for richness and evenness. Sampling sites were pooled into *high* (rectangle), *intermediate* (circle), and *low* (triangle) complexity datasets.
(TIF)

S2 Fig Determination of optimal granularity. Running time analysis for distributing different number of sequence pairs to 5 and 10 cluster nodes.
(TIF)

S1 Text XML configuration file example and description. The properties of CLUSTOM-CLOUD application are defined into “clustom.xml” file.
(PDF)

S2 Text The CLUSTOM-CLOUD manual. The introduction, system requirements, configuration and usage are described.
(PDF)
