# Supplementary figures and images for: CLUSTOM-CLOUD: In-Memory Data Grid-Based Software for Clustering 16S rRNA Sequence Data in the Cloud Environment
Source: PLoS One. 2016 Mar 8;11(3):e0151064. doi: 10.1371/journal.pone.0151064 (PMC4783016; doi:10.1371/journal.pone.0151064)

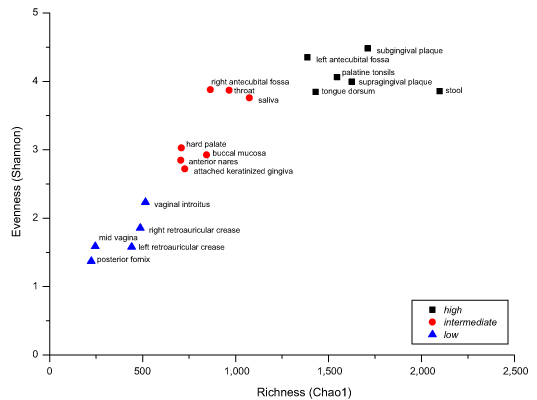

Supplement: S1 Fig — 16S rRNA sequence data from 18 human body sites was analyzed for richness and evenness. Sampling sites were pooled into high (rectangle), intermediate (circle), and low (triangle) complexity datasets. (TIF) [file pone.0151064.s001.tif]

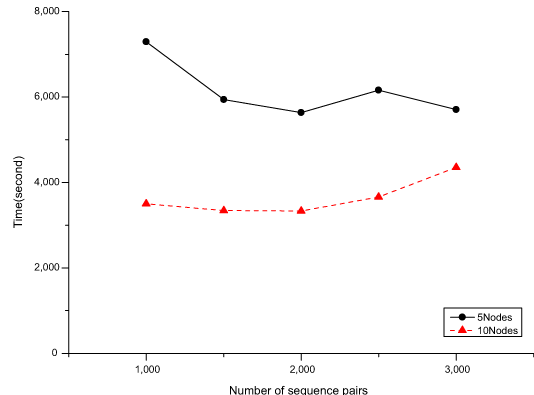

Supplement: S2 Fig — Running time analysis for distributing different number of sequence pairs to 5 and 10 cluster nodes. (TIF) [file pone.0151064.s002.tif]
