# Supplementary material for: CLUSTOM-CLOUD: In-Memory Data Grid-Based Software for Clustering 16S rRNA Sequence Data in the Cloud Environment
Source: PLoS One. 2016 Mar 8;11(3):e0151064. doi: 10.1371/journal.pone.0151064 (PMC4783016; doi:10.1371/journal.pone.0151064)
Supplement: S1 Text — The properties of CLUSTOM-CLOUD application are defined into “clustom.xml” file. (PDF) [file pone.0151064.s003.pdf]

```xml
<?xml version="1.0" encoding="UTF-8"?>
<!DOCTYPE properties SYSTEM "http://java.sun.com/dtd/properties.dtd">
<properties>
	<comment>CLUSTOM PROPERTIES</comment>
	<entry key="imdg_server">127.0.0.1:5701</entry>
	<entry key="imdg_ip_range">127.0.0.*</entry>
	<entry key="imdg_ip_member">127.0.0.1</entry>
	<entry key="imdg_port_number">5701</entry>
	<entry key="the_number_of_threads">8</entry>
	<entry key="chunk-size">2000</entry>
	<entry key="aws-enabled">false</entry>
	<entry key="access-key"></entry>
	<entry key="secret-key"></entry>
	<entry key="region"></entry>
	<entry key="host-header"></entry>
</properties>
```

Property	Description
imdg_server	A compulsory node IP of IMDG server.
imdg_ip_range	An interface IP range to IMDG server.
imdg_ip_member	All IMDG members IP.
imdg_port_number	A port number of each IMDG node.
the_number_of_ threads	The number of threads.
chunk_size	data size for allocating to each node (the number of sequence pairs)

aws-enabled(optional)	Choose it true if you want to use the Amazon Web Service environment.
access-key(optional)	An access key of your AWS.
secret-key(optional)	A secret key of your AWS.
region(optional)	The region name of your AWS.
host-header(optional)	A host header of your AWS.
