# Supplementary material for: CLUSTOM-CLOUD: In-Memory Data Grid-Based Software for Clustering 16S rRNA Sequence Data in the Cloud Environment
Source: PLoS One. 2016 Mar 8;11(3):e0151064. doi: 10.1371/journal.pone.0151064 (PMC4783016; doi:10.1371/journal.pone.0151064)
Supplement: S2 Text — The introduction, system requirements, configuration and usage are described. (PDF) [file pone.0151064.s004.pdf]

CLUSTOM-CLOUD

v1.0 (October 14, 2015)

In-Memory Data Grid-based software for clustering large scale 16S rRNA sequence data in the cloud environment

INDEX

1 INTRODUCTION

2 SYSTEM REQUIREMENTS

2.1 Minimum System Requirements

2.2 Recommended System Requirements

2.3 OS Supports

3 Configuration

3.1 Pre-requisition

3.2 clustom.xml

3.3 clustom-env.sh

3.4 servers.conf

3.5 SSH login without password

4 HOW TO USE CLUSTOM-CLOUD?

4.1 Arguments

4.2 How to use CLUSTOM-CLOUD

4.2.1 Startup CLUSTOM-CLOUD Server

4.2.2 Startup CLUSTOM-CLOUD Client

1 INTRODUCTION

This document is written for introducing CLUSTOM-CLOUD and explaining how to use it. After reading this document, the user is able to:

- configure CLUSTOM-CLOUD configuration files
  (clustom.xml, clustom-env.sh, servers.conf).
- startup/shutdown servers and clients.

2 SYSTEM REQUIREMENTS

The CLUSTOM-CLOUD is implemented in JAVA and In-Memory Data Grid (Hazelcast 3.3) version which is network sensitive. So, gigabit network or higher environment is recommended. The system requirements are listed below.

2.1 Minimum System Requirements

	Master Node	Worker Node
CPU Cores	1	1
Memory	4 GB	1 GB
Network	1Gbit Ethernet	1Gbit Ethernet
Supported OS	CentOS, UBUNTU, Windows 7, OSX 10.9.5	
Java version	above 1.7.0_71	

2.2 Recommended System Requirements

	Master Node	Worker Node
CPU Cores	8	4
RAM	16 GB	16 GB
Network Speed	1/10 Gbit Ethernet	1/10 Gbit Ethernet
Supported OS	CentOS 6.4, UBUNTU 12.x,Windows7,OSX 10.9.5-10.11	
Java version	above 1.7.0_71	

2.3 OS Supports

If you have installed Java Runtime Environment (JRE) on your machine, you can use CLUSTOM-CLOUD. We have tested on CentOS 6.4, Ubuntu 12.x, OSX 10.9.5-10.111 and Windows 7. Although OS is not specified, we recommend using Linux or OSX. If you are using Windows OS, please install the Cygwin first.

3 CONFIGURATION

CLUSTOM-CLOUD is implemented in JAVA and it does not use any other executable program inside, so it can be running on any kind of operating system without requiring other external programs to be installed.

3.1 Pre-requisition

To execute CLUSTOM-CLOUD, please make sure the Java Runtime Environment (JRE) be installed on your computer. Put the configuration file clustom.xml(default configuration file) and shell-script files on all the nodes so that they can act correctly. You can download with following site.

- JRE http://www.oracle.com/technetwork/java/javase/downloads/index.html.
- CLUSTOM-CLOUD http://clustom_cloud.kribb.re.kr

3.2 clustom.xml

The properties of CLUSTOM-CLOUD application are defined into "clustom.xml" file. Each property and description are listed blow.

Property	Description	IsRequired?
imdg_server	CLUSTOM-CLOUD representative node's IP address.<br>It's needed to join the cluster as a computing node.<br>e.g.) 192.168.1.1:5701	Yes
imdg_ip_range	Setting CLUSTOM-CLOUD cluster's ip range. It support wild character such like '*'<br>e.g.) 192.168.*.*, 192.*.*.*, *.*.*.*	Yes
imdg_ip_member	listing all node's IP addresses<br>e.g.) 192.168.1.1, 192.168.1.2, 192.168.1.3,192.168.1.4	Yes
imdg_port_number	Setting port number<br>5701	Yes
the_number_of_KMERDISTANCE_threads	Setting the thread number for *k*-mer distance operations	Yes
the_number_of_NW_local_threads	Setting the thread number for Global Alignment Operations	Yes
chunk-size	Setting the chunk size for *k*-mer distance operation and Global Alignment operation. (default value is 2000)	Yes

The flowing snippet is sample clustom.xml for single mode.

```
<?xml version="1.0" encoding="UTF-8"?>
<!DOCTYPE properties SYSTEM "http://java.sun.com/dtd/properties.dtd">
<properties>
	<comment>CLUSTOM-CLOUD PROPERTIES</comment>
	<entry key="imdg_server">127.0.0.1:5701</entry>
	<entry key="imdg_ip_range">127.0.0.*</entry>
	<entry key="imdg_ip_member">127.0.0.1</entry>
	<entry key="imdg_port_number">5701</entry>
	<entry key="the_number_of_KMERDISTANCE_threads">8</entry>
	<entry key="the_number_of_NW_local_threads">8</entry>
	<entry key="chunk-size">2000</entry>
	<entry key="aws-enabled">false</entry>
	<entry key="access-key"></entry>
	<entry key="secret-key"></entry>
	<entry key="region"></entry>
	<entry key="host-header"></entry>
</properties>
```

3.3 clustom-env.sh

The variables for CLUSTOM-CLOUD application are defined into "clustom-env.sh" file. Each variable name and description are listed blow.

Property	Description
JAVA_HOME	Setting installed java directory path<br>e.g.) /usr/java/jre_1.7.71
CLUSTOM_HOME	Setting CLUSTOM-CLOUD home directory path<br>e.g.) /home/clustom
CC_HEAPSIZE	Setting Java heap memory size.
CC_OFFHEAPSIZE	Setting Java Off Heap memory size.

Note) If you have 4GB computer memory, assign 3GB to CC_HEAPSIZE and the remained 1GB to CC_OFFHEAPSIZE.

3.4 servers.conf

servers.conf file should include list of working servers. And you have to explicitly put ip address of servers to it. The following snippet is the example code.

```
192.168.1.2
192.168.1.3
192.168.1.4
…
```

3.5 SSH login without password

CLUSTOM-CLOUD uses OpenSSH for communication among cluster node. And to skip frequent authentication phase, it would be better to register ssh key to nodes.

First log in on *Node1* as user *clustom* and generate a pair of authentication keys. Do not enter a passphrase:

```
[clustom@Node1:~]$ ssh-keygen -t rsa
Generating public/private rsa key pair.
Enter file in which to save the key (/home/clustom/.ssh/id_rsa):
Created directory '/home/ clustom /.ssh'.
Enter passphrase (empty for no passphrase):
Enter same passphrase again:
Your identification has been saved in /home/ clustom /.ssh/id_rsa.
Your public key has been saved in /home/ clustom /.ssh/id_rsa.pub.
The key fingerprint is:
3e:4f:05:79:3a:9f:96:7c:3b:ad:e9:58:37:bc:37:e4 clustom@Node1
```

Now use ssh to create a directory ~/.ssh as user *clustom* on *Node2*. (The directory may already exist, which is fine):

```
[clustom@Node1:~]$ ssh clustom@Node2 mkdir -p .ssh
clustom@Node2's password:
```

Finally append *clustom's* new public key to clustom@Node2:.ssh/authorized_keys and enter *clustom*'s password one last time:

```
[clustom@Node1:~]$ cat .ssh/id_rsa.pub | ssh clustom@Node2 'cat
>> .ssh/authorized_keys'
clustom@Node2's password:
```

From now on you can log into *Node2* as *clustom* from Node1 without password:

```
[clustom@Node1:~]$ ssh clustom@Node2
```

Note. Depending on your version of SSH you might also have to do the following changes:

Put the public key in .ssh/authorized_keys2
Change the permissions of .ssh to 700
Change the permissions of .ssh/authorized_keys2 to 640

4 HOW TO USE CLUSTOM-CLOUD?

4.1 Arguments

The CLUSTOM-CLOUD executable jar clustom-cloud.jar supports the following options.

1) Cluster Node side ; Server-Side

Option name	Short Name	Description	Usage
ConfigFile	-c	Configuration for CLUSTOM-CLOUD environment<br>Default configuration file is clustom.xml	-c clustom.xml

2) Client-Side

Option name	Short Name	Description	Usage
ConfigFile	-c	Configuration for CLUSTOM-CLOUD environment	-c clustom.xml
GlobalAlignmentCutOff	-g	Global alignment cut-off threshold	-g 0.03
RandomSampleSize	-r	Random sample size to determine *k*-mer distance cut-off threshold	-r 3000
InputFile	-i	Input file	-i HMP_V1_V3-L450_500.fasta
DuplicationFastaFilter	-d	On/Off duplication sequences filter	-d true

4.2 How to use CLUSTOM-CLOUD

This chapter explains how to startup and shutdown application.

4.2.1 Startup CLUSTOM-CLOUD Server

You may have the configured files (*clustom.xml, clustom-env.sh, servers.conf*).

This sample assumes the variables as blow

```
export JAVA_HOME=/Library/Java/JavaVirtualMachines/jdk1.7.0_71.jdk/Contents/Home
export CLUSTOM_HOME=/home/clustom_cloud/clustom_cloud
export CC_HEAPSIZE=3G
export CC_OFFHEAPSIZE=1G
```

Startup server(s).

```
[clustom@Nod1:~]$ bin/clustom.sh server start

Or

[clustom@Nod1:~]$ bin/start-all.sh
```

You can see the text as blow

```
[INFO] ClustomCloud run with CC_HEAPSIZE 1G

[INFO] ClustomCloud run with CC_OFFHEAPSIZE 3G

ClustomCloud Server (2434) has been started...
```

Shutdown server(s).

```
[clustom@Nod1:~]$ bin/clustom.sh server stop

Or

[clustom@Nod1:~]$ bin/stop-all.sh
```

You can see the text as blow

```
[INFO] ClustomCloud run with CC_HEAPSIZE 1G
[INFO] ClustomCloud run with CC_OFFHEAPSIZE 3G
ClustomCloud Server 2434 has been stopped
```

4.2.2 Startup CLUSTOM-CLOUD Client

After successfully starting servers, you can start client for executing clustom task. Actually starting the client is same as executing clustom task. The following snippet is sample command line for test.

- Input File: /home/clustom/data/sample.fasta
- GlobalAlignmentCutoff : 97%
- RandomSampleSize : 1000

The following snippet is the sample command to execute clustom.

```
[clustom@Nod1:~]$ bin/clustom.sh client start –i data/sample.fasta –r 1000 –g 0.03
```

The result will be located in directory whose name is same as inputfile name.
